# The development of a new oral health patient reported outcome measure: the New South Wales public dental services approach

**DOI:** 10.1186/s41687-024-00777-x

**Published:** 2024-08-19

**Authors:** Rebecca Chen, Shilpi Ajwani, Bradley Christian, Claire Phelan, Ravi Srinivas, Josephine Kenny, Mark O’Connor, Kara Clarke, Woosung Sohn, Albert Yaacoub

**Affiliations:** 1 Westmead Applied Research Centre, Faculty of Medicine, and Health, The University of Sydney, 176 Hawkesbury Rd, Westmead, NSW 2145 Australia; 2 Faculty of Medicine and Health, Sydney Dental School, The University of Sydney, Sydney, Australia; 3 Sydney Local Health District, Sydney, NSW Australia; 4 South Eastern Sydney Local Health District, Kogarah, NSW Australia; 5 South Western Sydney Local Health District, Liverpool, NSW Australia; 6 Western Sydney Local Health District, Westmead, NSW Australia; 7 Illawarra Shoalhaven Local Health District, Nowra, NSW Australia; 8 Centre for Oral Health Strategy, The Ministry of Health, Sydney, NSW Australia; 9 Nepean Blue Mountains Local Health District, Penrith, NSW Australia; 10 School of Nursing and Midwifery, Western Sydney University, Liverpool, NSW Australia

## Abstract

**Background:**

Addressing Patient Reported Outcomes (PROs) is essential for patient-centred care, shared decision making and improved health outcomes. Value-based health care systems in New South Wales (NSW) have a growing focus on collecting and using PROs that matter most to patients to improve their healthcare outcomes. Developing oral health patient reported outcomes measures (OH-PROM) is a first step towards value-based oral health care. This paper describes the development process of an adult and child OH-PROM tool that can be piloted for NSW public dental patients.

**Methods:**

An expert panel was assembled to undertake a systematic process of developing OH-PROMs for NSW Health. Key methodological considerations included: (1) forming an expert panel to specify the target population and context of implementation, (2) rapid literature review and environmental scan to identify existing validated OH-PROM tools for adults and children. (3) consensus gathering with the expert panel (4) consumer feedback, and (5) finalisation of the tool for electronic oral health record (eOHR) integration to establish a set of questions, that were relevant, context-appropriate, and important to oral healthcare outcomes for patients using public dental services.

**Results:**

The panel considered a total of 59 questions from two child (15), and four adult (44) Oral Health Related Quality of Life (OHRQoL) questionnaires used to collect OH-PROMs. These questions were mapped to the four key dimensions of OHRQoL for OH-PROMs: *Oral Function, Orofacial Pain, Orofacial Appearance, and Psychosocial Impact*. The consensus resulted in seven questions that aligned with these four dimensions to form two new NSW OH-PROM tools: one for adults and one for children. The tools were tested with consumers for understandability and usefulness before being incorporated into the electronic oral health record system, in readiness for future pilot testing.

**Conclusion:**

The process for developing new OH-PROMs for NSW public dental services took a pragmatic approach that combined literature appraisal, expert consensus, and consumer consultation. Future work will assess the implementation of the OH-PROM tool and test its validity for broader use as an outcome measure for value-based oral healthcare.

## Introduction

Globally, healthcare systems see a necessity to adopt value-based healthcare systems to deliver the best healthcare outcomes to patients in a financially sustainable way [1]. With rising healthcare demands, an aging population and an increasing burden of complex non-communicable diseases, understanding what patients and communities value most is a foundational step to more effective and efficient care [1]. Patient-reported outcome measures are a crucial way to understand the patient’s perspective and measure this in a systematic way. By understanding the patient, we shift our health system paradigms from “What is the matter with people” to “What matters to people” [1]. A recent systematic review of value-based healthcare systems internationally has highlighted the variability and lack of well-implemented PROM measures [2]. This paper has called for flexible frameworks to guide the uptake of value-based care and developing PROMs allowing for localisation and flexibility of its application to health conditions and contexts [2]. In the Australian context, the National Health Reform Agreement 2020–2025 highlighted the use of PROMs in clinical care to empower patients to be more involved in their healthcare [3]. The Australian Commission on Safety and Quality in Healthcare has defined PROMs as “any report of the status of a patient’s health condition that comes directly from the patient, without interpretation of the patient’s response by a clinician or anyone else” [4, 5].

In the dental setting, recent systematic reviews specific for Patient Reported Outcome Measures for dental patients have identified four dimensions that are essential components of a patient’s oral health experience namely: *Oral Function, Orofacial Pain, Orofacial Appearance and Psychosocial Impacts* [6, 7]. These essential components should be considered when developing dental specific PROMs. The International Consortium of Health Outcomes Measurement (ICHOM) in collaboration with large public dental organisations internationally have also developed an Adult Oral Health Standard Set (AOHSS) which includes some questions that are reported directly from the patient [8]. Jurisdictions who have contributed to the consortium have also highlighted the benefits of OH- PROMs to deliver consistent, best practices, and reduce unwarranted variation to enable the quantification of value generated for the patient and system [9, 10]. The use of routinely collected and tailored PROM tools can improve patient/provider communication, ensures quality and enables the health system to measure oral health patient-driven healthcare outcomes [11–13].

New South Wales (NSW) Health is the public health provider for Australia’s largest state. In the NSW Health context, there is a system-wide transition to emphasise value-based healthcare with concentrated efforts to implement a broad range of generic and condition-specific PROMs adjacent to service delivery [14]. In alignment with this, NSW public dental services are also strategically moving towards a value-based oral health system. A structured and localised process to collect and use OH-PROMs in routine oral healthcare is the foundational building block to drive value-based oral health service delivery within NSW Health. This study is the first part of a broader program of work aiming to implement OH-PROMs in NSW public dental services. The aim of the first stage of the program is to develop a standardised tool for adult and child patients to record OH-PROMs within NSW public dental services.

## Methods

### Phases of development

The development of a standardised tool comprised multiple phases that occurred concurrently. The general phases of our work included: (1) forming an expert panel to specify the target population and context of implementation, (2) rapid literature review, (3) consensus gathering with the expert panel (4) consumer feedback, and (5) finalisation of the tool for eOHR integration.

### Phase 1 Forming an expert panel to specify the target population and context

The expert panel was formed to ensure a pragmatic end-to-end design and implementation of an OH-PROM embedded into clinical practice within NSW public dental services. An expression of interest was circulated to all 15 Local Health Districts (LHDs) dental services across NSW. Six LHDs confirmed their participation. It was essential to include key stakeholders within each of the six LHDs who could lead and influence the implementation of OH-PROMs in NSW public dental services. Thus, the expert panel consisted of six dental directors/public oral health specialists, two policy officers from the Centre for Oral Health Strategy at the NSW Ministry of Health and two senior public oral health clinicians with academic affiliations. Members had varied dental clinical backgrounds including dentists, public dental health specialists, and oral health therapists.

The expert panel identified the target population and context of implementation being patients who were seeking generalist care within NSW Health public dental clinics. Public dental services in NSW are delivered across 15 Local Health Districts (LHDs) and two speciality networks. The services have over 600 dental and oral health clinicians who provide dental care across approximately 175 public dental facilities in NSW. NSW public dental services offer a range of services to children and adults who meet eligibility criteria [15]. They also have a prioritisation criterion for emergency situations, as well as for patient groups most in need and at the highest risk of dental diseases [16]. The type of dental treatment ranges from episodic emergency care to comprehensive general dental treatment and specialist care. As this PROM project was in its initial stage of the broader Value-based oral health program the expert panel determined that only patients receiving general dental treatment would be targeted.

### Phase 2 Rapid evidence review

The development of the OH-PROMs process began with a rapid literature review and environmental scan of the evidence, including a search of peer-reviewed literature and grey literature (including policy documents and white papers from the policy registers like the Analysis & Policy Observatory in Australia, and National Institute for Health and Care Excellence in the UK, as well as further email communications with authors of these papers jurisdictions) to identify existing information related to OH-PROMs. The review focused on evidence specific to dental patient reported outcome measures that were published in the main electronic databases including PubMed and Medline over the last 30 years. Whilst there is a paucity of published evidence reviews on the utilisation of OH-PROMs, there were existing examples of health services in The Netherlands [17] and the US [18] that have used validated OHRQoL questionnaires to collect OH-PROMs [6, 7]. Thus, our rapid literature review also leveraged existing systematic reviews that identified OHRQoL measures that have been used for general OH-PROM collection [6]. Dimensions identified by this existing systematic review namely: *Oral Function, Orofacial Pain, Orofacial Appearance and Psychosocial Impact* would form part of our inclusion criteria [7]. Additionally, the Australian Commission on Safety and Quality in Health Care (ACSQHC) guided our search by identifying existing OHRQoL tools specific to oral health [4] as well as highlighting the need to consider the emerging work of the International Consortium for Health Outcome Measures’ (ICHOM) Adult Oral Health set of questions [8].

Inclusion and exclusion criteria for the literature search developed by the panel were pragmatically chosen specific to the NSW Health public dental service context of implementation (Table 1). The panel agreed that our OH-PROM should be mapped to the OHRQoL dimensions identified by previous literature [6]. The OH-PROM for public dental services also needed to be quick to administer (20 questions or less), clinically relevant and simple to understand [17]. The panel agreed that there were different nuances when developing OH-PROMS for the child (under 18 years) and adult (18 years and above), therefore the literature review was split and run in parallel for adults and children.

**Table 1:** Inclusion and exclusion criteria of existing OH-PROM instruments

Inclusion criteria	Exclusion criteria
Oral Health related PROMs or OHRQoL measure	Condition-specific or used in in-patient setting
In English	Setting-specific (e.g., measures that were used in residential aged care facilities)
20 questions or less	Extensive discharge tools used in hospitals
Developed with a process outlined in the peer-reviewed literature or previously validated.	No clear process outlined.
Addressed three or more of the OHRQoL dimensions: *Oral Function, Orofacial Pain, Orofacial Appearance*, and *Psychosocial Impact*	Did not address OHRQoL dimensions.

### Phase 3 Consensus gathering with the expert panel

Once the expert panel decided on the context of implementation, they were provided an additional template to reach consensus around the (A) relevance to OHRQoL dimensions (B) consolidation of the questions and (C) comparable dimensions between the adult and child set. This template also had a list of questions from tools that fit the inclusion and exclusion criteria mapped to the OHRQoL dimensions. A parallel process to review the list of questions was conducted for the adult and child sets. All our expert panel members had worked in the fast-paced, high-demand services with public dental services that aimed to reach priority populations. This ensured relevance and practical implementation aspects were considered. We also reviewed the inclusion of these questions to fit the previously published and identified dimensions of OHRQoL [6]. The expert panel ensured that the consolidation of questions between the child and adult set also had comparable dimensions.

### Phase 4 Consumer feedback

Consumer feedback was paramount for the development of OH-PROMs and is embedded in NSW Health processes for developing new patient-centred models of care [19]. Consistent with this, consumer feedback was sought from active oral health consumers from the participating LHDs. Firstly, questions were placed on the Sydney Health Literacy Lab’s Health Literacy editor, generating a grade 6 reading level, which is lower than the recommended grade 8 reading level for health literacy documents [20]. Consumers were then given a printed version of the draft OH-PROM questions and were asked about whether the questions were (1) easy to understand and (2) if they were comfortable answering the questions as well as the opportunity to provide further feedback on these measures.

### Phase 5 Finalisation of the tool for eOHR integration

After consumer feedback was sought the expert panel reviewed the feedback before finalising the questions. These were then formatted into a templated questionnaire that would be embedded into the current electronic Oral Health Record (eOHR) system currently used by NSW public dental services.

## Results

The results of our paper will be presented in the five phases of our work outlined in the methods section of our paper.

### Phase 1 Specifying the target population context of implementation

The expert panel first discussed the context for implementation [11]. Agreement on the context and the target population guided the discussion about the specific questions and system requirements for the OH-PROMs. The agreed upon setting and process for OH-PROM collection is outlined in Table 2.

**Table 2:** Specified context for the implementation of pilot OH-PROMs in the NSW Health context

Who is the target patient population?
The target population for OH-PROM collection was identified as adults and children using NSW public dental services for general (comprehensive) dental treatment. Those requiring episodic urgent, emergency or specialist care were not included in the initial pilot. The expert panel chose this context because these patients would be starting a course of care that required general comprehensive treatment planning and would be more likely to engage in the collection of OH-PROMs for shared decision making.
**When and how often would OH-PROMs be collected?**
It was decided to specify longitudinal time points for data collection to allow the patient, clinician, and health service to compare and measure the change in the OH-PROMs. The OH-PROMs would be collected at the start of the first appointment (the baseline PROM), and again at the end of the course of care (the discharge PROM). In some cases, where the full benefit of the dental care that has been received is not fully appreciated at the point of discharge (e.g., denture care), a reflective OH-PROMs (the follow-up PROM), would be collected approximately 3 months post discharge. This is particularly relevant for patients receiving dentures who may not experience the full benefits of their care for several weeks or months after their final dental appointment. The follow-up process also provides an opportunity for patients to reflect on their OH-PROMs and receive further advice or care if needed.
**How would the OH-PROMs be collected?**
The expert panel strategically chose to integrate the collection of the OH-PROMs with the eOHR system (Titanium®) used within NSW public dental services to support to uptake and the efficiency of OH-PROMs collection. The OH-PROMs questionnaire would be completed at the point of care by the clinicians interviewing the patients/carers whilst recording the results directly in the eOHR. The eOHR allows current and previous records of OH-PROMs to be available at future appointments, supporting patient-clinician communication and improving the provision of patient centred care. The expert panel also agreed to provide an alternative option for some patients to complete a paper-based OH-PROMs in the waiting room. This option provides patients/carers with the opportunity to make use of their time in the waiting room. This pre-filled paper form would be provided to the clinician once they are in the clinical setting. The questions on the paper form are identical to the electronic form on the eOHR. For young children, parents could act as a proxy to report the OH-PROMs on behalf of their child. Older children (14 years and older) have the option of answering the questions by themselves. The variations in who answered the questions and how the OH-PROM was collected were recorded for future evaluation purposes.

### Phase 2 Results of the rapid evidence review

The rapid literature review was conducted specific to the target population and the context of implementation. The review identified two child (with a total of 15 questions), and four adult tools (with a total of 44 questions) that fit the inclusion/exclusion criteria. This included a set of questions from another Australian health jurisdiction that had applied OH-PROMs based on the ICHOM measures [8]. The working group mapped each of these questions to the four Oral Health Related Quality of Life dimensions found in Table 3.

**Table 3:** Adult and Child PROM tools mapped to the Oral Health Quality of Life dimensions (OHRQoL)

OHRQoL tool [abbreviation of tool] [ref]	Total number of questions	Year published	Oral Health Related Quality of Life (OHRQoL) dimensions
*Adult PROM instruments*			
Oral Health Impact Profile-14 [OHIP-14] [21]	14	1997	• Oral Function • Orofacial Pain • Psychosocial Impact • Orofacial Appearance
Oral Health Impact Profile-5 [OHIP-5] [22]	5	2016	• Oral Function • Orofacial Pain • Psychosocial Impact
ICHOM’s Adult Oral Health Standard Set [AOHSS] [8]	18	2021	• Oral Function • Orofacial Pain • Orofacial Appearance • Psychosocial Impact
Other Australian jurisdiction—grey literature	7	Not published	• Oral Function • Orofacial Pain • Orofacial Appearance • Psychosocial Impact
*Child PROM instruments*			
Scale of Oral Health Outcomes for 5-year-old children [SOHO-5] [23]	7	2012	• Oral Function • Orofacial Pain • Orofacial Appearance • Psychosocial Impact
Child Oral Impacts on Daily Performances [C-OIDP] [24]	8	2006	• Oral Function • Orofacial Appearance • Psychosocial Impact

### Phase 3 Consensus gathering with the expert panel

#### Relevance of the questions

**Fig. 1:**
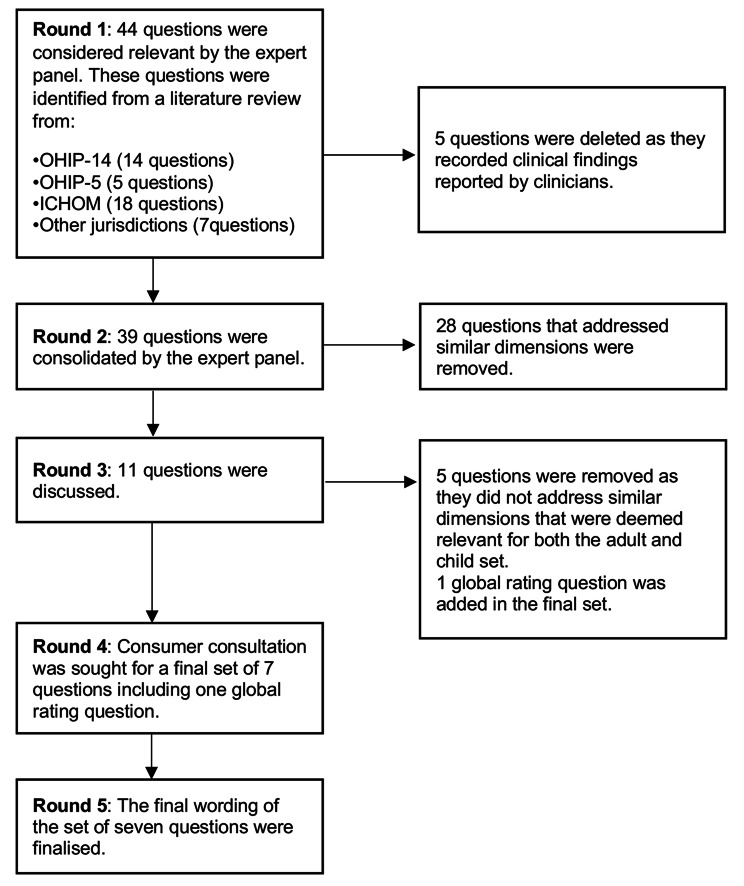
Flow chart of the development process for the adult set

**Fig. 2:**
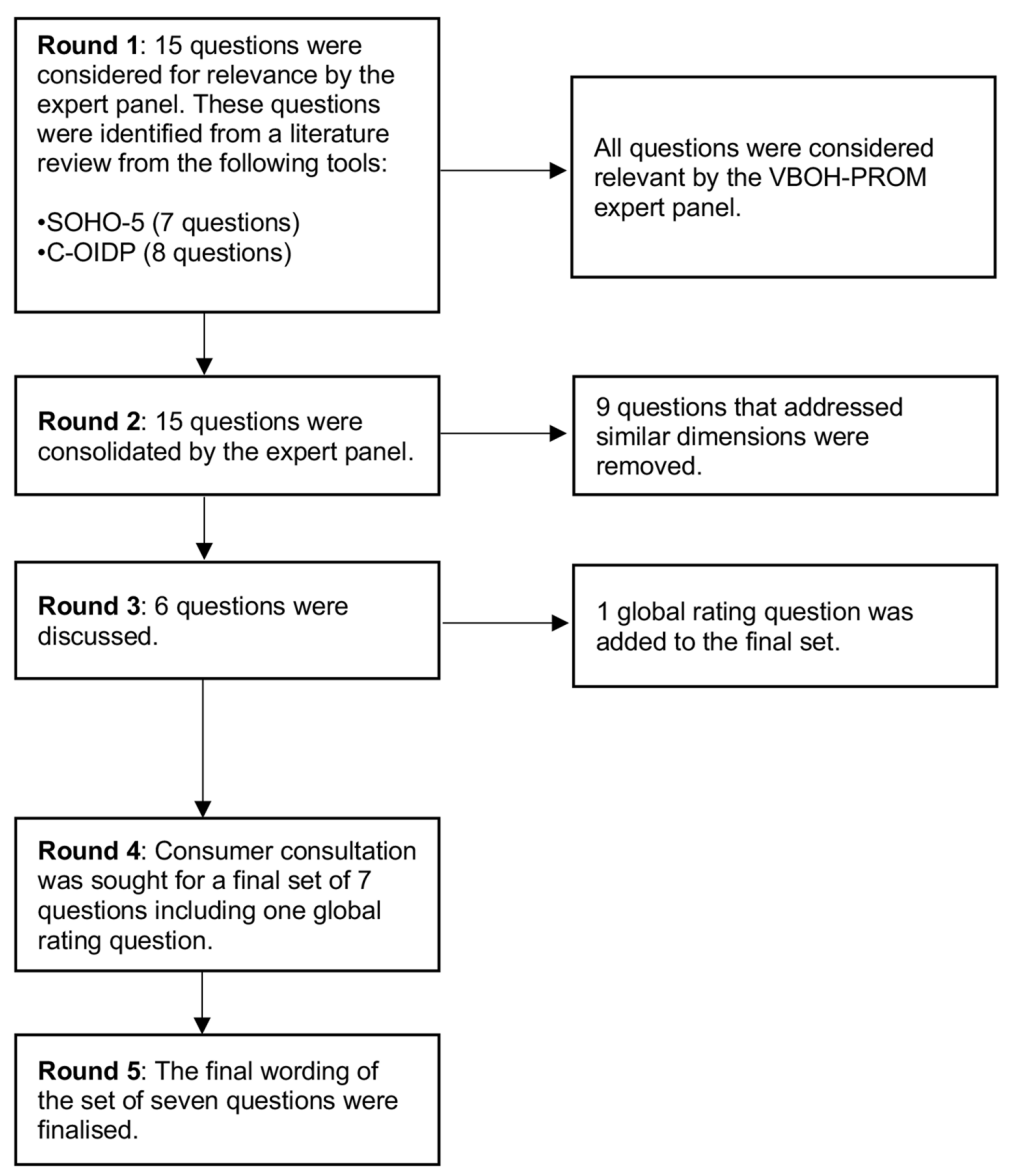
Flow chart of the development process for the child set

Of the initial 44 adult questions considered by the expert panel (Fig. 1) 39 questions were mapped and considered relevant to an OHRQoL dimension. Five questions were removed from the ICHOM Adult Oral Health Standard Set (AOHSS) [8], as they were findings that were reported by clinicians. This did not fit the Australian Commission on Safety and Quality in Healthcare’s definition of a PROM, which is stated as “a report of the patient’s conditions that comes directly from the patient” [4, 5]. Of the 15 child questions that were presented to the group, all questions were considered relevant to the OHRQoL dimensions and fit the criteria for a OH-PROM (Fig. 2).

#### Consolidation of the questions

At this stage, the group focused on consolidating all the questions that addressed similar OHRQoL dimensions. Many of the 39 questions in the adult set addressed similar dimensions and were consolidated into 11 questions. For the child set, 15 questions were consolidated to six questions.

#### Comparable dimensions for adults and child OH-PROM tools

Ensuring comparable dimensions between child and adult OH-PROMs is crucial for establishing system-wide measures that could drive future quality improvement projects [11]. Therefore, only questions addressing similar OHRQoL dimensions were selected with a final set of six questions for each tool. The expert panel also agreed that a global oral health rating question should be included, resulting in consensus for a final set of seven questions (inclusive of the global rating question) for the adult and child tool.

### Phase 4 Results of the consumer feedback

We obtained 47 anonymous responses (25 for the adult and 22 for the child set) from active oral health consumers from the participating LHDs. The feedback on whether each of the questions was easy to understand, showed that respondents rated 86–96% of the questions positively. When considering whether they were comfortable answering the questions, positive responses for the questions ranged from 88 to 98%. The only feedback from consumers regarding the wording of the questions related to the ambiguity of the term *“usual activities”*. In response to this, the expert panel revised the term *“usual activities”* to *“daily activities”* resulting in the following question *“Because of the condition of your mouth, teeth and gums how often do you have difficulty doing daily activities?”.* Some consumers also sought clarity about whether questions like “*Because of the condition of your mouth, teeth and gums do you feel embarrassed?”* related specifically to the patient’s oral health or were also inclusive of their general health conditions. This feedback informed implementation strategies including training materials focused on how staff could ask these questions specific to the oral health condition of the patient. After considering consumer feedback, the expert panel finalised the two sets of questions (Tables 4 and 5).

**Table 4:** POH-PROMs core set of questions for adults mapped to the OHRQoL dimension

Question	OHRQoL dimension
Global rating question 1. How is the health of your mouth, teeth and gums?	Global rating
Because of the condition of your mouth, teeth and gums…	
2. How often do you have trouble eating?	Oral Function
3. How often do you find is difficult to speak clearly?	Oral Function
4. How often do you have trouble sleeping?	Orofacial Pain
5. How often do you have difficulty doing daily activities?	Psychosocial Impact
6. How often do you feel embarrassed?	Orofacial Appearance
7. How often do you have pain?	Orofacial Pain

**Table 5:** POH-PROMs core set of questions for children mapped to the OHRQoL dimension

Question	OHRQoL dimension
Global rating question 1. How is the health of your child’s mouth, teeth and gums?	Global rating
Because of the condition of your child’s mouth, teeth and gums…	
2. How often do they have trouble eating?	Oral Function
3. How often do they have trouble drinking?	Oral Function
4. How often do you have trouble speaking clearly?	Oral Function
5. How often do you have trouble sleeping?	Orofacial Pain
6. How often do you feel difficulty doing daily activities?	Psychosocial Impact
7. How often do they avoid smiling?	Orofacial Appearance

### Phase 5 Finalisation and eOHR integration

In the next stage, the two finalised OH-PROM tools were built into the eOHR as electronic forms (eForms). The eForms incorporated fields to also record variability in the way the OH-PROMs were completed, e.g., whether the OH-PROM was completed by the patient in the waiting room or by direct interview with the dental clinician. For the child PROMs, our eForm also recorded who was answering the questions for the child OH-PROMs, i.e., the child, their parents/carers, or others. This was considered important for future analysis of the implementation process.

## Discussion

Internationally, the recent shifts towards value-based oral health care have highlighted the importance of systematically using PROMs to ensure that the patient voice remains core to oral health service delivery. This has prompted many health systems internationally and in Australia to consider integrating dental PROMs into oral health clinical service delivery. An essential component of developing PROMs is to seek consumer input at the onset of development in a meaningful way [25]. However, there is paucity in the literature about how this can be pragmatically applied to larger public oral health contexts. Our paper aims to share the methods and pragmatic lens that large multi-site public health service units have undertaken to develop OH-PROMs. By engaging key clinical experts and ensuring shared decision making our process bridges the implementation gaps; thus expediting the translation of evidence into practice. Our expert panel was able to balance the learnings from the academic literature, and a variety of settings internationally to localise the OH-PROMs tools for use in public dental services in Australia. Our paper builds on systematic reviews [6, 26, 27] and existing localised projects from the Netherlands [17], the US [6] and other jurisdictions within Australia including Victoria [28]. Our core set of seven questions for an adult and child OH-PROMs tool aligns with the four (OHRQoL) dimensions including *Oral Function, Orofacial Pain, Orofacial Appearance, and Psychosocial Impact* that have previously been synthesised from international sources[6, 26]. Our paper demonstrates how local input, including consumer consultation during the development of OH-PROMs ensures that the questions are understandable and appropriate to support broad implementation of OH-PROMs. The involvement of key working party members with a strong understanding of the eOHR (Titanium®) system also enabled the OH-PROMs to be embedded as an eForm, reducing barriers to implementing and scaling the project in the future [29]. Although developed for the Australian context, our methods are transferrable and bridge an implementation science gap by providing a framework for other public health organisations that run safety-net clinical services nationally and internationally seeking to integrate OH-PROMs into their service delivery.

This phase of the project demonstrates the necessary first step to enable the widespread collection and use of OH-PROM data to support clinician-patient communication and system-level data to improve patient-centred care [11, 30]. To facilitate the timely collection of OH-PROMs, our project also strategically set out structural components including the development of eForms within the centralised eOHR system used across NSW public dental services. Previous literature has indicated that a major barrier to the clinician’s engagement with the collection of PROMs often related to the lack of interoperability of the PROM collection tool with their regular electronic record database [29, 31]. Cognisant of this, the expert panel chose to integrate the OH-PROMs with the eOHR before the pilot project, to support clinician uptake by enabling the efficient collection of OH-PROMs [30].

There are some limitations with our tool as it is currently only in English. The questions may be challenging to understand for non-English speaking patients. However, these patients will have access to interpreter services within NSW public dental services who will assist them in completing the OH-PROMs. Future iterations of this tool may involve the translation to popular languages reflective of our diverse patient populations.

Our focus on pragmatic data collection directly from the patient’s perspective has also meant that we may not have the granularity compared to other tools like ICHOM’s 18 questions in the AOHSS and 14 questions in the OHIP-14. Furthermore, compared to the ICHOMs questions, the NSW OH-PROMs are all collected directly from the patient and our tool does not have any clinical questions that can only be determined by clinicians. However, as our tool is mapped to all four dimensions of OHRQoL: *Oral Function, Orofacial Pain, Orofacial Appearance*, and *Psychosocial Impact* [6], it continues to enable comparison to other OH-PROMs used in the literature [27]. This alignment to the four quality of life dimensions could support future implementation and comparison of non-dental specific PROMs broadly used across the NSW Health system [11]. This may include a comparison of dimensions to the Patient Reported Outcome Measurement Information System (PROMIS) [32]. PROMIS is a set of person-centred measures that evaluates and monitors physical, mental, and social health in adults and children [32]. Alignment to other PROMs like PROMIS used in the NSW Health system could be important for future service planning especially when these tools have been extended to include economic analysis [33].

This quality improvement project laid the foundational work to develop and describe the process for designing a set of OH-PROMs. Value-based oral health emphasises the importance of capturing the patient’s perspective on their own health outcomes, to measure improvements in receiving care. Further work is needed to develop and evaluate the implementation of the OH-PROMs tools across NSW public dental services. This will ensure the broad reliability and validity of this tool across different settings including different iterations such as the translation of the tool into different languages. Future pilot projects with robust evaluations will inform the scale-up and broad uptake of OH-PROMs in NSW public dental settings.

## Conclusion

This paper details the approach to develop patient reported outcome measures for use in NSW public dental services. The development process ensured a balance between scientific rigour, expert input, and the consumers’ perspective. A comprehensive framework is being designed to evaluate and validate these OH-PROM tools to ensure system scalability, and the potential of these tools to measure quality improvement projects in alignment with value-based oral health care. Future widespread use and systematic collection of OH-PROMs provide consumers, dental practitioners and policymakers with a consistent measure of value-based oral health care.

## Data Availability

Data sharing is not applicable to this article as no datasets were generated or analysed during the current study.
